# A Comparative Study on the Ferroelectric Performances in Atomic Layer Deposited Hf_0.5_Zr_0.5_O_2_ Thin Films Using Tetrakis(ethylmethylamino) and Tetrakis(dimethylamino) Precursors

**DOI:** 10.1186/s11671-020-03301-4

**Published:** 2020-04-07

**Authors:** Baek Su Kim, Seung Dam Hyun, Taehwan Moon, Keum Do Kim, Young Hwan Lee, Hyeon Woo Park, Yong Bin Lee, Jangho Roh, Beom Yong Kim, Ho Hyun Kim, Min Hyuk Park, Cheol Seong Hwang

**Affiliations:** 1 Department of Materials Science and Engineering and Inter-University Semiconductor Research Center, Seoul National University, Seoul, 08826 Republic of Korea; 2 School of Materials Science and Engineering, Pusan National University, 2 Busandaehak-ro-63beon-gil, Geumjeong-gu, Busan, 46241 Republic of Korea

**Keywords:** Ferroelectric Hf_0.5_Zr_0.5_O_2_ film, Atomic layer deposition, Metal-organic precursor, Wake-up phenomenon, Interfacial layer

## Abstract

The chemical, physical, and electrical properties of the atomic layer deposited Hf_0.5_Zr_0.5_O_2_ thin films using tetrakis(ethylmethylamino) (TEMA) and tetrakis(dimethylamino) (TDMA) precursors are compared. The ligand of the metal-organic precursors strongly affects the residual C concentration, grain size, and the resulting ferroelectric properties. Depositing Hf_0.5_Zr_0.5_O_2_ films with the TDMA precursors results in lower C concentration and slightly larger grain size. These findings are beneficial to grow more ferroelectric-phase-dominant film, which mitigates its wake-up effect. From the wake-up test of the TDMA-Hf_0.5_Zr_0.5_O_2_ film with a 2.8 MV/cm cycling field, the adverse wake-up effect was well suppressed up to 10^5^ cycles, with a reasonably high double remanent polarization value of ~40 μC/cm^2^. The film also showed reliable switching up to 10^9^ cycles with the 2.5 MV/cm cycling field without involving the wake-up effect but with the typical fatigue behavior.

## Introduction

Atomic layer deposited Hf_1-*x*_Zr_*x*_O_2_ (HZO, *x* ~ 0.5) thin films have been the leading contender as the ultra-thin ferroelectric (FE) layer in the field of semiconductor devices for memory and logic applications. This is because the fluorite-structure FE HZO film can be scaled down even below 10 nm, and homogeneously deposited on three-dimensional nanostructures by utilizing matured atomic layer deposition (ALD) techniques. In addition, it is compatible with the conventional TiN electrode [1, 2], which can hardly be achieved from the conventional perovskite-structure ferroelectrics. Despite the significant improvement in the HZO film processing and device fabrication using the ALD-based thin films in the past years, there are several unresolved shortcomings. Especially, the reliability of the fluorite-structure ferroelectrics is uncertain. Currently, the wake-up effect and the limited number of endurance are considered the most severe issues [3]. Generally, the polarization–electric field (P–E) curves are pinched in the pristine state, suggesting that the coercive field (E_c_) is spatially non-uniform, and several FE domains are pinned [4]. After electric field cycling with a field strength higher than E_c_, more symmetric and square-like P–E curves can be achieved, a phenomenon known as a wake-up effect. In some cases, such a wake-up process goes for 10^4^–10^5^ cycles, which is a typical endurance cycle of ca. flash memory, making the device and system operation complicated [5]. The limited number of endurance is another critical issue if it is intended to be used as working memory (endurance > 10^15^ is required). For metal-ferroelectric-metal capacitor structure, the maximum reported endurance is less than 10^11^ [6], and for metal-ferroelectric-semiconductor gate-stack in ferroelectric field-effect-transistor, the endurance is limited up to 10^5^ times [3, 7].

Various origins of the wake-up effect were suggested in the literature. The suggested mechanisms are pinning of domain boundaries by defects, such as impurities, oxygen vacancies, and presence of the non-ferroelectric phase (cubic or tetragonal phase) at the interfaces adjacent to the electrodes or semiconductor channel in the pristine state [5, 8–10]. The pinning site concentration is expected to decrease during the repetitive polarization switching. Also, electric field cycling transforms the interfacial tetragonal or cubic phases into the FE orthorhombic phase [5]. This study mainly focused on improving the electrical performances of the HZO film or eliminating the wake-up effect by adopting an alternative Hf and Zr precursors during the ALD process, which may result in a lower impurity concentration, especially carbon impurity.

For the ALD processes using the metal-organic precursors, it is almost inevitable to induce residual impurities, such as C, N, and H in the grown film, which are most probably originated from the organic ligands. Kim et al. [11, 12] showed that by changing the deposition temperature of HfO_2_ and HZO film, the polymorphism and resulting electrical properties could be controlled. From Auger electron spectroscopy (AES), the C concentration in ALD HZO thin film increased with decreasing deposition temperature, which might result from the imperfect ligand exchange reactions [11, 12]. Also, the lateral grain diameter decreased with increasing C concentration. The formation of the unstable or metastable phases (tetragonal, orthorhombic, and cubic) rather than the stable monoclinic phase in such fluorite structure films is closely related with the grain size effect [13–16]. Thus, controlling the impurity concentration is crucial in achieving the desired phase (FE orthorhombic) as well as enhancing the electrical reliability of the film.

For the ALD of FE HZO thin films, the most frequently used metal-organic Hf and Zr precursors are the tetrakis[ethylmethylamino]hafnium (TEMAH) and tetrakis[ethylmethylamino]zirconium (TEMAZ) [11, 12, 17]. These precursors were developed for the metal-organic chemical vapor deposition with the intention of facile ligand decomposition via the electric charge transfer between the methyl and ethyl groups [18–20]. However, this type of thermally induced ligand decomposition and subsequent removal of the (fragments of) organic ligands adversely interfere with the facile ALD reaction resulting in the impurity (C, H, and N) incorporation in the film [11, 17–20].

In contrast, the tetrakis[dimethylamino]hafnium (TDMAH) and tetrakis[dimethylamino]zirconium (TDMAZ) precursors, which have also been used to deposit the FE HZO films [21–24], have only methyl groups in their ligands. Therefore, such an adverse effect might not be serious, although the complete suppression of the thermal decomposition cannot be guaranteed.

This study performed a comparative analysis between the HZO films grown by the ALD processes with two different metal precursors; TEMAH/TEMAZ and TDMAH/TDMAZ. The latter process resulted in the lower C concentration in the film, which significantly improved the electrical performance of the HZO film. Under the optimized switching cycling conditions, almost no wake-up effect was achieved while the switchable polarization remained at ~ 40 μC/cm^2^.

## Methods/Experimental

### Preparation of the Hf_0.5_Zr_0.5_O_2_ Thin Films

This work examined the influence of types of metal-organic precursors on the structure and electrical performances of the atomic layer-deposited Hf_0.5_Zr_0.5_O_2_ thin films. The HZO thin films were deposited using a 4-inch-diameter scale thermal ALD reactor with TDMAH (or TEMAH), TDMAZ (or TEMAZ) and ozone (190 g/m^3^ concentration) as the Hf precursor, Zr precursor, and oxygen source, respectively. The optimized ALD process with TEMAH/TEMAZ precursors were as in the authors’ previous studies [5, 9, 11–16]. The HZO thin films with TDMAH/TDMAZ precursors were prepared by thermal ALD at a substrate temperature of 260 °C. A Hf:Zr ratio of 50:50 was chosen for the electrical test, since the composition has been reported to show the largest remanent polarization (P_r_) value in previous studies [17, 25, 26]. The HZO thin films with the TDMAH/TDMAZ precursors were deposited with 1:1 ALD cycle ratio of Hf and Zr precursors on TiN/Ti/SiO_2_/Si substrate. One ALD cycle was composed of source feeding (2 s) - source purge (20 s) -ozone feeding (3 s) - ozone purge (10 s) process. The growth rate of the HZO film was 0.13 nm per cycle and the 10-nm-thick HZO thin films were prepared by TDMAH/TDMAZ precursors for the experiments. The optimum conditions may vary depending on the volume of the ALD chamber. Table 1 shows the comparison of physical properties of the TEMA and TDMA sources. The TiN (50 nm) and Ti (5 nm) films were deposited using sputtering with a sputtering power of 5 kW on the thermally oxidized p-type Si substrates using a commercial sputtering tool (Endura, Applied Materials). The deposited HZO films are only partially crystalline or amorphous in the as-deposited state, so the subsequent annealing for crystallization was conducted using a rapid thermal process (RTP) at 450 °C in N_2_ atmosphere.

**Table 1:** Comparison between TDMA-Hf, Zr and TEMA-Hf, Zr source specification

	Melting point (°C)	Temp (0.1Torr; °C) [17]	Temp (1Torr; °C) [17]	State (@canister temp; °C)	Vaporization type	Note
TDMAH	30 [17]	48	75	Liquid (70)	Vapor pressure	Fast exhaustion
TDMAZ	60 [17]	49	77	Liquid (70)	Vapor pressure	Fast exhaustion
TEMAH	− 50 [27]	83	113	Liquid (50)	Bubbler	
TEMAZ	− 20 [28]	76	106	Liquid (50)	Bubbler	

### Characterization of the Chemical/Physical Properties of the Hf_0.5_Zr_0.5_O_2_ Thin Films

The crystalline structures of the deposited films were analyzed using an X-ray diffractometer (XRD, X’pert pro, Panalytical) within a grazing incidence geometry with an incidence angle of 0.5°. The microstructures of the samples were analyzed using a scanning electron microscopy (SEM, S-4800, Hitachi), and the grain size distribution was analyzed using a Gwyddion software [29] through a watershed method. The chemical compositions of the deposited HZO film was analyzed using X-ray fluorescence (XRF, Quant’X, Thermo SCIENTIFIC), and the in-depth variations in chemical compositions, including impurities such as C, were analyzed using a time-of-flight Auger electron spectroscopy (AES, PHI-700, ULVAC-PHI).

### Characterization of the Electrical Properties of the Hf_0.5_Zr_0.5_O_2_ Thin Films

To analyze the electrical properties of the HZO films, the top TiN electrodes were reactively deposited through a sputtering process with a power of 100 W under the 92.6%-Ar/7.4%-N_2_ atmosphere. The TiN top electrodes were patterned using a shadow mask with circular holes having a diameter of 300 μm. The P–E characteristics were analyzed using a ferroelectric tester (TF analyzer 2000, Aixacct systems) at a measuring frequency of 1 kHz. The endurance test was conducted with rectangular bipolar pulses with heights and width of 2.8 ~ 3.8 MV/cm and 10 μs, respectively, generated by a pulse generator (81110A, Agilent) and a ferroelectric tester (TF analyzer 2000, Aixacct systems). The capacitance–voltage (C–V) characteristics were measured using an impedance analyzer (4194A, Hewlett-Packard) under the sinusoidal AC pulses with a frequency of 10 kHz and a height of 50 mV combined with DC bias. The dielectric constants of HZO films were calculated from the measured capacitance as well as electrode area measured by optical microscopy and thickness measured using spectroscopic ellipsometry (ESM-300, J. A. Woollam). The current density-electric field (J-E) characteristics were analyzed using a semiconductor parameter analyzer (4155B, Hewlett-Packard) under DC bias with a delay time of 1 s.

## Results and Discussion

**Fig. 1:**
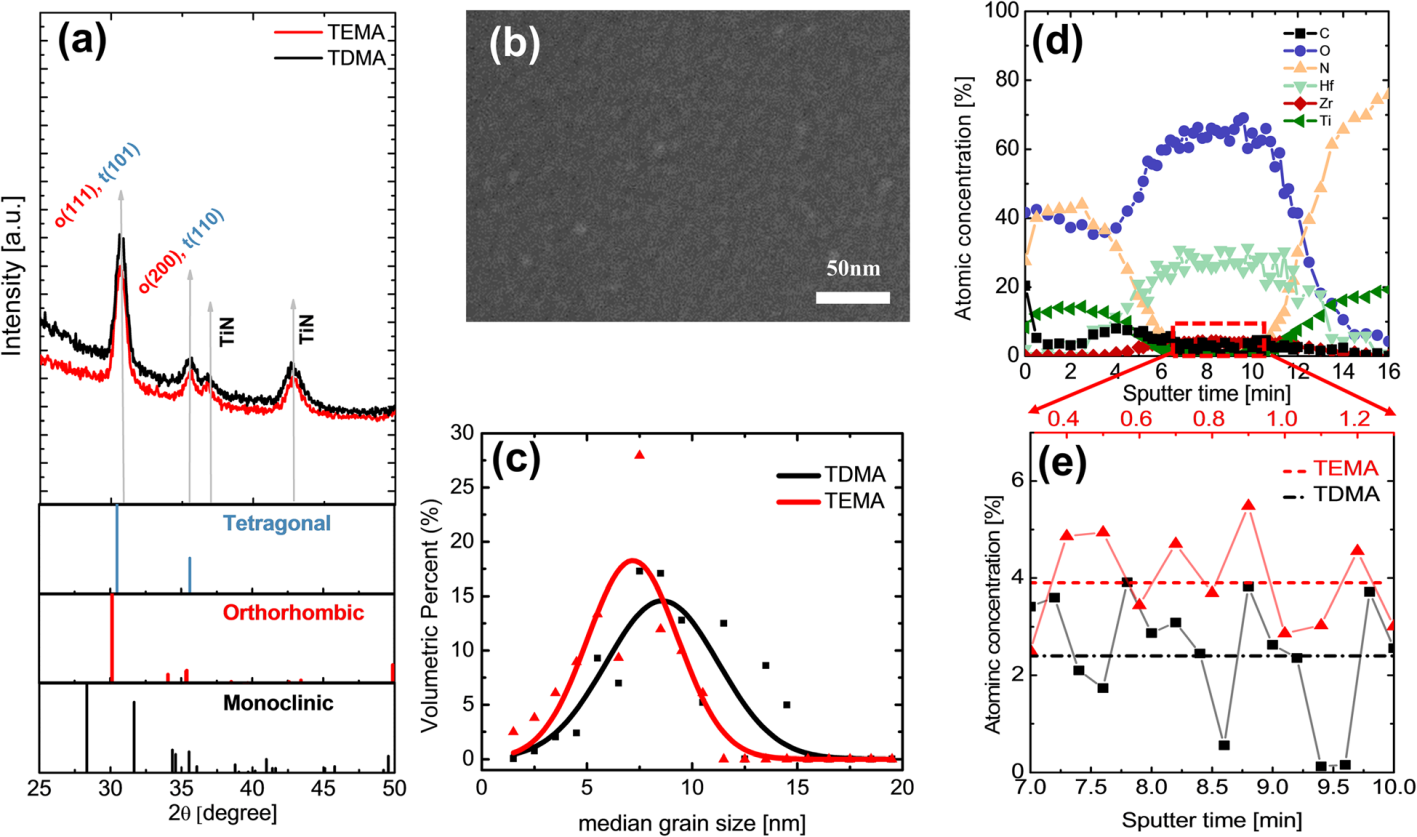
**(a)** GIXRD patterns of HZO thin films deposited using TDMAH/TDMAZ (black curve) and TEMAH/TEMAZ (red curve), the reference patterns taken from literature for the monoclinic, tetragonal, and orthorhombic phases are attached in the bottom panels. (**b)** The planar SEM image of TDMA HZO films and (**c)** the grain size distribution of TEMA (red; reproduced from Ref. [15]), TDMA (black) HZO. (**d)** The depth profile of the TDMA HZO thin films analyzed using time-of-flight Auger electron spectroscopy, and (**e)** the enlarged low concentration region of the HZO film part. Average C concentration of TDMA (black dash-dot line), TEMA (red dash line; reproduced from Ref. [12]), HZO is presented

Figure 1a shows the grazing incidence X-ray diffraction (GIXRD) patterns of 10-nm-thick Hf_0.5_Zr_0.5_O_2_ thin films deposited using TDMAH/TDMAZ (TDMA-HZO, black curve) and TEMAH/TEMAZ (TEMA-HZO, red curve) with an incidence angle of 0.5°. The reference patterns taken from literature for the monoclinic, tetragonal, and orthorhombic phases are appended in the bottom portion. From both GIXRD patterns of the TDMA and TEMA HZO films, the intensities of the diffraction peaks from the monoclinic phase were negligible and no notable difference in the peak shapes and intensities could be identified. Thus, no significant differences in the crystallographic structure between TDMA and TEMA HZO was experimentally confirmed from GIXRD.

The microstructure, including lateral grain size, is another critical factor that can strongly affect the FE properties of HZO thin films [13–16, 30]. Thus, the microstructures of the TDMA and TEMA HZO films were analyzed using scanning electron microscopy (SEM). Figure 1b shows the planar SEM image of TDMA HZO films. Various previous studies reported that the HZO thin films deposited using thermal ALD showed columnar grain structure, suggesting that the vertical grain size is as large as the film thickness [1, 5, 11, 31]. The distribution of the lateral grain size analyzed using the above-mentioned software [29], was fitted with the Gaussian function. The grain size distribution of TEMA HZO (red curve) was taken from a previous study [15], and plotted with that of TDMA HZO (black curve) in Fig. 1c. As shown in Fig. 1c, the average lateral grain diameter of the TDMA HZO (8.5 nm) was larger than that of the TEMA HZO (7.1 nm). This could be the main reason for the improved FE performance of the TDMA HZO. According to previous reports, the formation of the metastable phases, such as orthorhombic and tetragonal phases, is driven by the kinetic origins, and the tetragonal and orthorhombic phases are preferred in the small grain size region [13, 16]. Much larger grains prefer to be monoclinic phases, smaller grain size prefers the tetragonal phase, and the slightly larger grain size prefers the orthorhombic phase. The almost overlap of the peak positions of the two phases (tetragonal and orthorhombic phases) in the diffraction patterns did not allow unambiguous identification of the major phase in the two films. However, the SEM and accompanying grain size analysis indicated that the TDMA HZO film could have a higher portion of the orthorhombic phase compared with the TEMA HZO film.

The different grain sizes could be originating as a result of the different level of the C-impurity concentration in the two films. The concentrations of impurities could strongly affect the microstructure and resulting ferroelectric properties of HZO thin films [11, 12, 32]. Therefore, the chemical composition of the TDMA and TEMA HZO thin films was analyzed using time-of-flight AES, and the resulting in-depth concentrations of various atoms such as Hf, Zr, O, C, Ti, and N in TDMA HZO film were plotted as a function of sputtering time in Fig. 1d. Figure 1e shows the enlarged low concentration region of Fig. 1d (red dashed box) in the HZO film part. The average C concentration in TDMA HZO film (black square) was ~ 2.4%, which is ~ 38% smaller than that (~ 3.9%) of TEMA HZO film (red triangle) [12], of which AES data were reported in the authors’ previous study [12]. All other concentrations, including N, did not show any notable differences.

Cho et al. suggested that the residual C impurities formed during the ALD process retarded the grain growth, and resulted in the small grain size of the finally deposited films [32]. A similar trend was observed for ferroelectric Hf_0.5_Zr_0.5_O_2_ thin films and pure HfO_2_ films by Kim et al. when the deposition temperature decreased from 280 to 200 °C [11, 12]. Jung et al. used computational simulations to show that the free energy difference between the tetragonal and monoclinic phase decreases with increasing C concentration in HfO_2_, suggesting that including C impurity enhances the stability of the metastable tetragonal phase [33]. Kuenneth et al. also examined the effect of C concentration on the free energy values of HfO_2_. However, they reported that the increase in C concentration did not result in the decrease of the free energy difference between the orthorhombic and monoclinic phase [34]. In Kuenneth et al.’s work, the substitutional C defects were considered, although the C impurities are generally known as interstitial defects in HfO_2_ [33, 35]. Therefore, the theoretical calculations did not clearly reveal that the C impurities could decrease the free energy difference between the tetragonal and orthorhombic phases. However, experiments have confirmed that the increase in C impurities could increase the tetragonal phase fraction in the ALD HZO thin films [11, 12, 33].

The lower C impurity concentration in the TDMA HZO film could be ascribed to the different thermal decomposition nature of the TDMA and TEMA ligands. The outmost carbon atoms in the TEMA ligands are prone to be thermally dissociated and remained on the film surface during the ALD process [11, 12], which may not be the case in TDMA ligand.

**Fig. 2:**
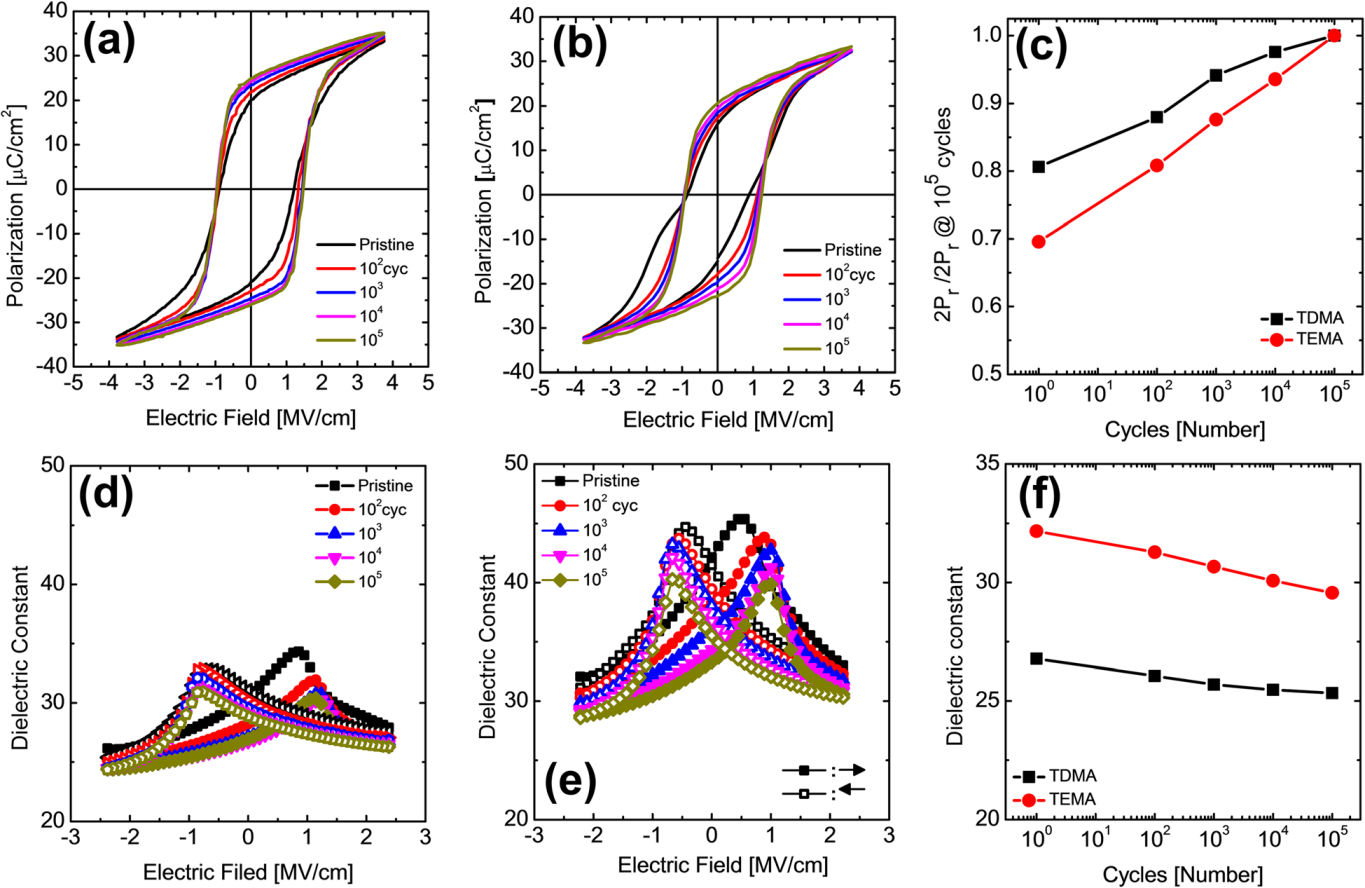
The polarization–electric field (P–E) curves of (**a**) the TDMA HZO and (**b**) TEMA HZO (reproduced from Ref. [5]) capacitors measured in pristine state and fatigued states pulsed by 10^2^, 10^3^, 10^4^, and 10^5^ times at 3.8 MV/cm-high and 10 μs-wide bipolar rectangular pulses. (**c)** The changes in 2P_r_ values of TDMA (black) and TEMA (red) HZO capacitors as a function of fatigue pulses. The dielectric constant–electric field curves of (**d**) the TDMA HZO and (**e**) TEMA HZO (reproduced from Ref. [5]) capacitors with the top and bottom TiN electrodes measured in pristine state and fatigued states. (**f)** The changes in dielectric constant values with increasing number of fatigue pulses for TDMA (black) and TEMA (red) HZO capacitors

As the next step, the effect of C concentration and resulting microstructure on the ferroelectric properties are discussed. Figure 2a and b show the P–E curves of MFM capacitors with the TDMA HZO and TEMA HZO films, respectively, measured in a pristine state and fatigued states switched by 10^2^, 10^3^, 10^4^, and 10^5^ times using the 3.8 MV/cm-high and 10 μs-wide bipolar rectangular pulses. From the P–E curves in Fig. 2a and b (pristine state), the P–E curve of TEMA HZO capacitor (black curve, Fig. 2b) is more strongly pinched in the pristine state compared to the TDMA HZO capacitor (black curve, Fig. 2a). Figure 2b clearly shows the humps in the pristine P–E curve of TEMA HZO capacitor, which is not the case for the pristine P–E curve of TDMA HZO capacitor. The humps in the P–E curve originates from the splitting of switching current peaks, which generally results from the spatial inhomogeneity in the internal electric field and/or coercive field. Figure 2c shows the changes in 2P_r_ values of TDMA and TEMA HZO capacitors as a function of fatigue pulses. The 2P_r_ values after 10^5^ times of electric pulses, compared to the pristine 2P_r_ values, of TDMA and TEMA HZO capacitors are ~ 80 and ~ 69%, respectively. This suggests that the TEMA HZO film has a higher wake-up behavior compared with the TDMA HZO film.

Figure 2d and e show the dielectric constant–electric field (ε_r_ - E) curves of the TDMA HZO and TEMA HZO capacitors measured in a pristine state and fatigued states switched by 10^2^, 10^3^, 10^4^, and 10^5^ times using the 3.8 MV/cm-high and 10 μs-wide bipolar rectangular pulses. The ε_r_ values of TDMA HZO capacitor are significantly lower than that of TEMA HZO capacitors at all test conditions. Figure 2f shows the changes in ε_r_ values with the increasing number of fatigue switching cycles for TDMA and TEMA HZO capacitors. The ε_r_ values were calculated by averaging ε_r_ values measured at the highest and lowest electric fields in Fig. 2d and e. From the GIXRD patterns in Fig. 1a, the monoclinic phase fractions in both films were negligible. Thus, the difference in ε_r_ value might be determined by the relative fractions of the tetragonal and orthorhombic phases or the defect concentration, which is expected to decrease the average dielectric constant with local lattice distortions. The ε_r_ value of the tetragonal phase (35–40) was higher than that of the orthorhombic phase (25–30). Thus, the high ε_r_ value of TEMA HZO capacitor indicates that it has a higher tetragonal phase fraction compared to the TDMA HZO capacitors. With the increasing number of fatigue pulses, the ε_r_ value of both TEMA HZO and TDMA HZO capacitors decreases, as shown in Fig. 2f. The magnitude of decrease in ε_r_ value during 10^5^ times polarization switching for TDMA HZO capacitor (26.8 to 25.3) was smaller than that for TEMA-HZO capacitor (32.2 to 29.6) by ~ 42%. This is consistent with the wake-up behavior shown in Fig. 2c.

The difference in tetragonal phase fraction and the resulting different ε_r_ value of TDMA and TEMA HZO thin films could be understood from the difference in C concentration. According to Kim et al. [12], the increase in C concentration decreases the free energy of the tetragonal phase compared to that of the orthorhombic phase (tetragonal phase is still more favorable compared with the orthorhombic phase). As a result, with the increasing C concentration, the tetragonal phase fraction is expected to increase. Since the C concentration of TDMA HZO film is lower than that of the TEMA HZO film, the tetragonal phase fraction in TDMA HZO film is expected to be lower than that of the TEMA HZO film. The difference in grain size shown in Fig. 1c also supports the same trend in relative crystalline phase fractions. According to Materlik et al. [30], the free surface energy of the tetragonal phase (2.5 J/m^2^) is lower than that (2.9 J/m^2^) of the orthorhombic phase, although these free surface energies were estimated to explain the experimental observations in HZO thin films with various thicknesses and Zr concentrations. Batra et al. [36] calculated the free surface energy of the various crystalline phases with various orientations and showed that the free surface energy of the tetragonal phase is lower than that of the orthorhombic and monoclinic phase. It is generally accepted that the high angle grain boundary energy is roughly 1/3 of the free surface energy [37]. Thus, the grain boundary energy of the tetragonal phase is the lowest compared with the orthorhombic and monoclinic phases, making it the most stable phase at the smallest grain size. These are consistent with the idea that the smaller grain size of the TEMA HZO tends to include a higher portion of the non-ferroelectric tetragonal phase compared with the TDMA HZO film, which had a larger average grain size. Therefore, the experimentally observed C concentration and grain size consistently supports the different crystalline structure and the resulting electrical properties of the TDMA and TEMA HZO thin films.

**Fig. 3:**
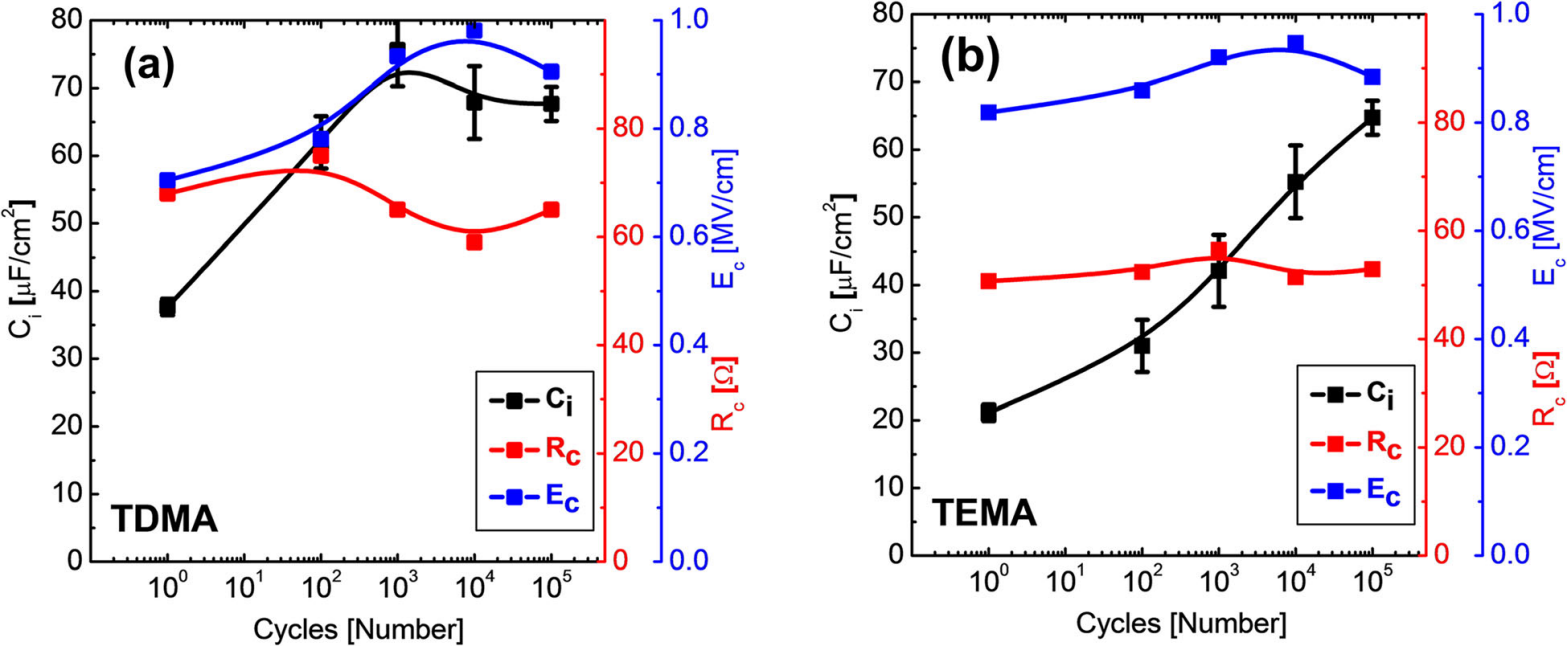
The changes of (**a**) TDMA HZO and (**b**) TEMA HZO (reproduced from Ref. [5]) in interfacial capacitance (C_i_), contact resistance (R_c_), and coercive field (E_c_) values with increasing number of fatigue pulses

To elucidate the mechanism behind the wake-up effect, the pulse switching measurement, which can estimate the interfacial capacitance (C_i_) originating from the non-ferroelectric layer near electrodes, was conducted on TDMA HZO and TEMA HZO capacitors [5]. Figure 3a and b show the changes in C_i_, contact resistance (R_c_), and E_c_ values with the increasing number of fatigue pulses for the TDMA HZO and TEMA HZO capacitors, respectively. The detailed measurement method and results are included in online Supporting Information. The data for TEMA HZO capacitor was taken from Kim et al.’s previous work [5], where the C_i_ value increases with the increasing number of electric field cycling [5]. In the pristine state, the C_i_ (37.6 μF/cm^2^) value of the TDMA capacitor is higher than that (21.1 μF/cm^2^) of the TEMA HZO capacitor by ~ 75%, suggesting that the thickness of the non-ferroelectric interfacial layer in TDMA HZO is much smaller than that in TEMA HZO film. On the other hand, the difference in E_c_ value in the pristine state of TDMA and TEMA HZO capacitors is only 13%, suggesting that the main reason for the difference in the pristine P–E characteristics of TDMA and TEMA HZO capacitors is the different thickness of the non-ferroelectric interfacial layers. Since R_c_ value is strongly affected by contact resistance for the electrical test setup, it may have lower importance compared to the other two factors. Therefore, the different P–E characteristics in the pristine state of TDMA and TEMA HZO capacitor could be consistently understood based on the previous wake-up model suggested by Kim et al. [5]. According to the previous studies, the oxygen vacancy concentration near the TiN electrodes is higher than that of the film bulk region in the pristine state. According to Hoffmann et al. [38], the increase in oxygen vacancy concentration enhances the stability of the tetragonal phase compared to that of the orthorhombic phase. During repetitive polarization switching in the endurance test, the interfacial tetragonal phase seemed to convert to the FE orthorhombic phase by diffusing out the oxygen vacancies into the bulk region of the film. The applied field also induced phase transition of the interfacial non-FE phase into the FE phase. Since the thickness of the interfacial layer of the TDMA-HZO capacitor is smaller than that of the TEMA-HZO capacitors in the pristine state, the wake-up effect during the field cycling could be mitigated.

**Fig. 4:**
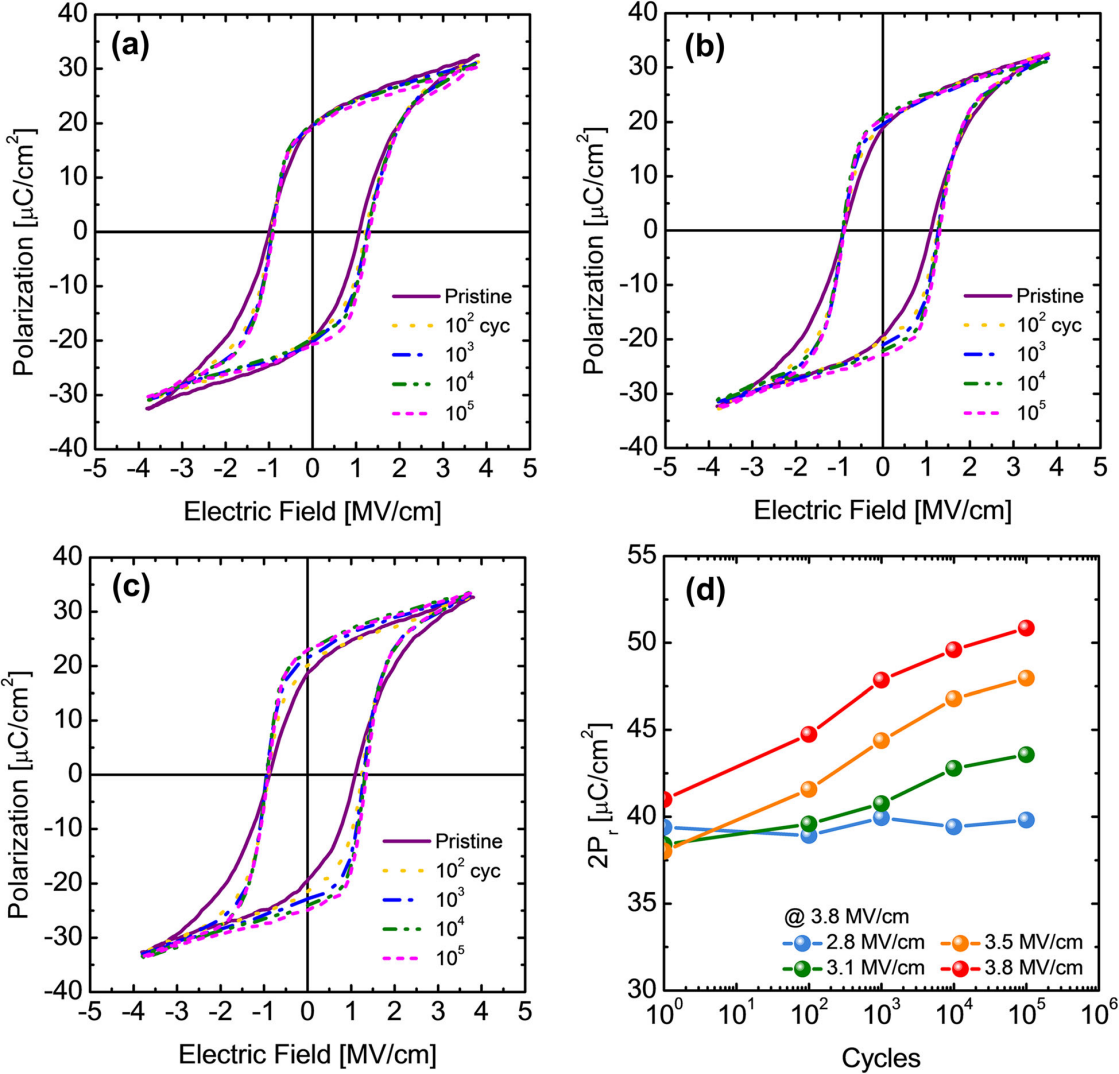
The polarization–electric field (P–E) curves measured during wake-up test with the fatigue pulse height of (**a**) 2.8, (**b**) 3.1, and (**c**) 3.5 MV/cm. (**d)** The changes in 2P_r_ value during the endurance test with 2.8, 3.1, 3.5, and 3.8 MV/cm-high fatigue pulses

Also, the amplitude of the fatigue pulse is another crucial factor that can strongly affect the wake-up phenomena of the fluorite-structure ferroelectrics [6, 8]. Therefore, the wake-up effect of TDMA HZO capacitor was examined using fatigue pulses with various amplitudes of 2.8, 3.1, 3.5, and 3.8 MV/cm. Figure 4a, b, and c show the P–E curves measured during the wake-up test with fatigue pulse heights of 2.8, 3.1, and 3.5 MV/cm, respectively. The changes in 2P_r_ during the wake-up test were summarized in Fig. 4d. Similar with the wake-up test result shown in Fig. 2a, the P–E measurement was conducted at the measuring electric field of 3.8 MV/cm, after a certain number of the wake-up cycles with the given field amplitude. The changes in P–E hysteresis decrease with decreasing amplitude of fatigue pulses as shown in Fig. 4a–c. Figure 4d shows a summary of the changes in 2P_r_ value during the endurance test with 2.8, 3.1, 3.5, and 3.8 MV/cm amplitude fatigue pulses. As seen in Fig. 4d, the magnitude of 2P_r_ increase after 10^5^ times of field cycling was 0.41, 5.18, 9.93, and 9.94 μC/cm^2^ for the different fatigue field amplitude, which correspond to ~ 1, ~ 13, ~ 26, and ~ 24% changes, respectively. This result implies that the wake-up effect is negligible when a fatigue pulse of 2.8 MV/cm amplitude was applied, where a reasonably high 2P_r_ value (~ 40 μC/cm^2^) could be still achieved.

The wake-up effect is strongly related to the drift of oxygen vacancies and the resulting phase transition from the tetragonal phase to the orthorhombic phase, mainly in the interfacial layer [9, 10]. The drift of oxygen vacancies should be strongly influenced by the amplitude of the fatigue pulses, and appropriately lower fatigue-test field amplitude (2.8 MV/cm in this case) can largely mitigate such an adverse effect. Although the achievable maximum 2P_r_ value was decreased from ~ 51 μC/cm^2^ (at 3.8 MV/cm) to ~ 40 μC/cm^2^ (at 2.8 MV/cm), ~ 40 μC/cm^2^ is still reasonably high value for ferroelectric memory devices. For the case of the TEMA HZO film, a similar strategy could be applied to mitigate the wake-up issue, but its initially low 2P_r_ value (~ 30 μC/cm^2^) could be the potential problem for such a method.

**Fig. 5:**
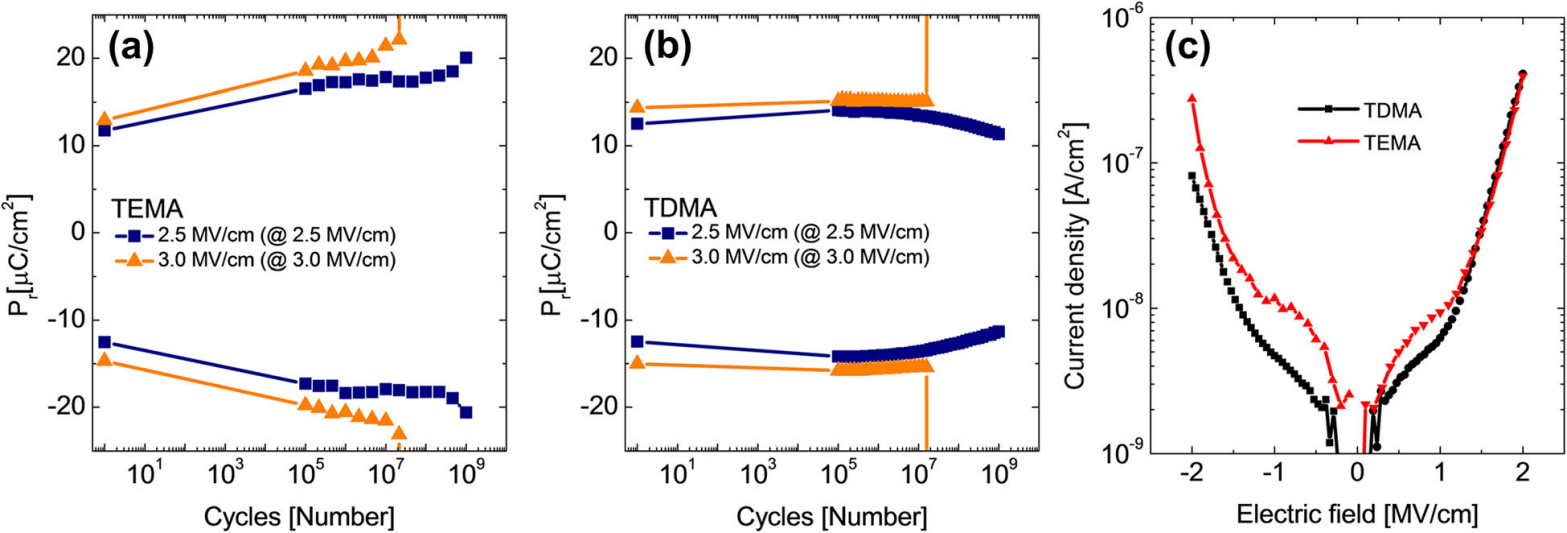
The results of endurance test of (**a**) TEMA HZO (reproduced from Ref. 39) and (**b**) TDMA HZO with the electric field cycling and pulse field amplitude of 2.5 and 3.0 MV/cm. The frequency of the rectangular double pulse for the endurance test was fixed at 100 kHz. (**c)** The current density–electric field curves of TDMA HZO (black) and TEMA HZO (red; reproduced from Ref. [39])

The influence of C concentration was further clarified by the endurance test up to 10^9^ cycles, as shown in Fig. 5a and b, which showed the variations in P_r_ under field amplitude of 2.5 and 3.0 MV/cm for the TEMA and TDMA HZO films, respectively. In both cases, the P_r_ values were estimated by the P–E loops with the maximum electric field of the identical strength to the cycling field, so the estimated P_r_ values are generally smaller than those values in Fig. 4, where the P–E tests were performed with 3.8 MV/cm. When the maximum field (3.8 MV/cm) for P–E test in Fig. 4 was utilized for the endurance tests, the films were early broken down, prohibiting the endurance tests up to the maximum cycle numbers. The two films showed consistent trends in the evolution of the P_r_ vs. cycle behavior: TEMA HZO film kept increasing the P_r_ values, whereas the trend was much lower for the case of the TDMA HZO film. The TEMA HZO film showed unsteady P_r_ changes before break down at ~ 10^7^ and ~ 10^9^ cycles using 3.0 and 2.5 MV/cm field cycling, respectively. In contrast, the TDMA HZO film showed no indication of breakdown up to ~ 10^7^ and ~ 10^9^ cycles at 3.0 MV/cm and 2.5 MV/cm field cycling, and sudden breakdown was observed. The P_r_ value decreased slightly after ~ 10^7^ under the cycling field strength of 2.5 MV/cm, which corresponds to the genuine fatigue behavior. Similar decay in the P_r_ performance with the switching cycles has been extensively reported for conventional perovskite ferroelectrics, which usually ascribed to the domain wall pinning by the increasing defect density [40, 41]. In the previous studies on the HZO-based ferroelectric thin films, such genuine fatigue behaviors have hardly been observed due to the involvement of significant wake-up and early breakdown, which was also the case in Fig. 5a. The data shown in Fig. 5b reveals that the HZO film may also suffer from the fatigue effect, known in the perovskite ferroelectric film, under the condition that the wake-up and early breakdown are appropriately addressed.

Figure 5c shows the comparison in the leakage current density performance of the two types of films. Due to the lower C concentration, TDMA HZO film had a lower leakage current than that of the TEMA HZO film at field strength < ~ 1.5 MV/cm, where the trap-assisted tunneling may dominate. As a result of the leakage current improvement in TDMA HZO, the film did not show the breakdown up to 10^9^ cycles, with relatively low field strength of 2.5 MV/cm.

However, in the higher field strength region, the difference becomes diminished, which may indicate that the high-field leakage current is more dominated by the interface Schottky barrier property, and such barrier property was less sensitive to the C concentration. Further research will be performed to more precisely identify the leakage current mechanism in subsequent work. The similar leakage currents in the high field region coincide with the no significant difference in the number of switching cycles before the breakdown at 3.0 MV/cm, shown in Fig. 5a and b.

## Conclusion

In conclusion, this work examined the influence of types of metal-organic precursors on the structure and electrical performances of the atomic layer-deposited Hf_0.5_Zr_0.5_O_2_ thin films. The adopted Hf and Zr precursors have either TEMA or TDMA ligands, where the former is more prone to the thermal decomposition compared to the latter. The ALD process using the precursors with TDMA ligands resulted in a lower C impurity concentration (~2.4 atomic % vs. ~3.9 atomic %) in the HZO film, which induced a slightly larger grain size (~8.5 nm vs. ~7.1 nm). As the slightly larger grain size prefers to have the ferroelectric orthorhombic phase rather than the non-ferroelectric tetragonal phase, the TDMA HZO film outperformed the TEMA HZO film, especially for the wake-up performance. When the wake-up field cycle was 2.8 MV/cm, the TDMA HZO film showed almost no wake-up effect, while a high 2P_r_ value of ~40 μC/cm^2^ could be achieved. This is significant merit over the severely waking-up property of the TEMA HZO film. The TDMA HZO film also contained a lower portion of the interfacial non-ferroelectric phase with the TiN electrodes, compared with the TEMA HZO film. Due to the lower C concentration, the TDMA HZO film showed a lower leakage current in the low field region (< ~1.5 MV/cm), but the high-field leakage current behaviors were similar. As a result, the number of switching cycles before breakdown was similar when the cycling field was as high as 3.0 MV/cm (~10^7^ cycles), but it could be extended over 10^9^ cycles when the cycling field was lower (2.5 MV/cm) for the case of the TDMA HZO film. The TDMA HZO film revealed that the typical fatigue behavior, i.e., decreasing P_r_ value with the increasing switching cycles, could be observed after ~ 10^7^ cycles at 2.5 MV/cm, which might be ascribed to the domain wall pinning by the accumulated defects, as for the conventional perovskite ferroelectric films.

## Supplementary information

**Additional file 1.** Pulse switching measurement for estimating the interfacial properties of Hf_0.5_Zr_0.5_O_2_ deposited using tetrakis (dimethylamino)hafnium and Zirconium precursors (additional file)

## Data Availability

The datasets supporting the conclusions of this article are included within the article and its Additional file 1.

## Abbreviations

TEMA: Tetrakis(ethylmethylamino)
TDMA: Tetrakis(dimethylamino)
TEMAH: Tetrakis[ethylmethylamino]hafnium
TEMAZ: Tetrakis[ethylmethylamino]zirconium
TDMAH: Tetrakis[dimethylamino]hafnium
TEMAZ: Tetrakis[dimethylamino]zirconium
TDMA HZO: Hf_0.5_Zr_0.5_O_2_ thin films deposited using TDMAH/TDMAZ
TEMA HZO: Hf_0.5_Zr_0.5_O_2_ thin films deposited using TEMAH/TEMAZ
ALD: Atomic layer deposition
AES: Auger electron spectroscopy
RTP: Rapid thermal process
XRD: X-ray diffractometer
GIXRD: Grazing incidence X-ray diffraction
SEM: Scanning electron microscopy
HZO: Hf_1-x_Zr_x_O_2_, x~0.5
FE: Ferroelectric
P_r_: Remanent polarization
E_c_: Coercive field
P–E: Polarization–electric field
C–V: Capacitance–voltage
J–E: Current density–electric field
C_i_: Interfacial capacitance
R_c_: Contact resistance
