# Supplementary material for: A Comparative Study on the Ferroelectric Performances in Atomic Layer Deposited Hf0.5Zr0.5O2 Thin Films Using Tetrakis(ethylmethylamino) and Tetrakis(dimethylamino) Precursors
Source: Nanoscale Res Lett. 2020 Apr 7;15:72. doi: 10.1186/s11671-020-03301-4 (PMC7138889; doi:10.1186/s11671-020-03301-4)
Supplement: Supplementary file 1 — Additional file 1. Pulse switching measurement for estimating the interfacial properties of Hf0.5Zr0.5O2 deposited using tetrakis (dimethylamino)hafnium and Zirconium precursors (additional file) [file 11671_2020_3301_MOESM1_ESM.docx]

**Supporting information for**

A comparative study on the ferroelectric performances in atomic layer deposited Hf_0.5_Zr_0.5_O_2_ thin films using tetrakis (ethylmethylamino) and tetrakis (dimethylamino) precursors

Baek Su Kim,^1,+^ Seung Dam Hyun,^1,+^ Taehwan Moon,^1^ Keum Do Kim,^1^ Young Hwan Lee,^1^ Hyeon Woo Park,^1^ Yong Bin Lee,^1^ Jangho Roh,^1^ Beom Yong Kim,^1^ Ho Hyun Kim,^1^ Min Hyuk Park,^2^* and Cheol Seong Hwang^1^*

1 Department of Materials Science and Engineering and Inter-University Semiconductor Research Center, Seoul National University, Seoul 08826, Republic of Korea

2 School of Materials Science and Engineering, Pusan National University, 2 Busandaehak-ro-63beon-gil, Geumjeong-gu, Busan 46241, Republic of Korea

+These authors equally contributed to this study.

*electronic mail: [minhyukpark@pusan.ac.kr](mailto:minhyukpark@pusan.ac.kr), [cheolsh@snu.ac.kr](mailto:cheolsh@snu.ac.kr)

**1. Pulse switching measurement for estimating the interfacial properties**

In the authors' previous studies, the interface properties of TEMA-HZO film change between the electrode and the ferroelectric (FE) thin film were evaluated by observing the transient switching current through pulse measurement [5]. In this work, the same measurements were performed for TDMA-HZO film to study how the interface characteristics change after wake-up field cycling. Details of this measuring method were also reported in the author’s previous work [5]. The pulse switching measurement process and the process of obtaining interfacial capacitance (C_i_), contact resistance (R_c_) and coercive field (E_c_) from this measurement were as follows.

The charging of the FE capacitor occurs in two steps:

i) The dielectric charging step.

At this step, FE switching does not occur because not enough electric field to FE switching is applied to the capacitor, and only dielectric charging occurs.

ii) The ferroelectric switching step.

When sufficient charging occurs, enough field is applied to the FE capacitor and the FE switching commences. When FE switching occurs, the FE ideally behaves like a resistor in the circuit. In real case, however, there is an additional interface layer that acts as a serially connected capacitor. Therefore, the FE and the interfacial layer form an RC circuit. In this case, the switching current is presented as Equation S1.

$I_{sw}\left( t \right)=I_{sw}^{0}e^{-\frac{t-t_{0}}{R_{L}C_{i}}} \left( t_{0}\leq t\leq t_{sw} \right)$ (S1)

, where t_0_, t_sw_, R_L_, and C_i_ are the time when ferroelectric switching starts, the time when the switching ends, the total resistance of the circuit and sample, and the interfacial capacitance, respectively. R_L_ is the summation of the internal resistance of the pulse generator (R_w_) and the digital oscilloscope (R_o_), the parasitic resistance (R_p_) and the contact resistance (R_c_). The summation of R_W_, R_O_ and R_P_ is estimated to be ∼104 Ω in this measurement setup. By measuring I_sw_ (t) over time and plotting log(I_sw_ (t)) vs t, R_L_C_i_ is obtained from the slope of the plateau region (t_0_ < t < t_sw_).

I_sw_^0^ is the current value at time when the switching starts, and is expressed as Equation S2.

$I_{sw}^{0}=\frac{\left( E_{appl}-E_{c} \right)d}{R_{L}}$ (S2)

, where E_appl_ is the applied field and d is the film thickness [5]. This means that at the moment of the FE film switching, E_c_, a portion of E_appl_ is applied to the FE layer while the remaining portion of the E_appl_ is applied over R_L_ [5]. Therefore, R_L_ and E_c_ can be calculated from the slope and the intercept of the x-axis by taking I_sw_^0^ at each E_appl_ and plotting the value as a linear function of E_appl_.

Figure S1 (a) is a schematic diagram of the process for measuring I_sw_(t). Before measuring I_sw_(t), the polarization should be poled to one direction by applying a write pulse having height of - 3.8 MV/cm and a length of 10μs. Then, I_sw_(t) was measured by applying a read pulse with E_appl_ (2.4, 2.9, 3.3, 3.8 MV/cm) and a length of 10μs. Figure S1 (b), (c), (d) show the I_sw_(t) in pristine state, after wake up cycling of 10^3^ and 10^5^, respectively. R_L_C_i_ was obtained from the slope of log(Isw(t)) vs. t. The I_sw_^0^, which is the current value at the beginning of the switching plateau, was extracted from Figure S1 (b) ~ (d) and plotted for E_appl_. R_L_ was calculate from the slope (=d/R_L_) of linear fitting. E_c_ was obtained from x-axis intercept. Figure S1 (e), (d) and (g) show the I_sw_^0^ vs. E_appl_ plot after pristine, 10^3^ and 10^5^ wake up cycling, respectively. C_i_ can be obtained from R_L_C_i_ / R_L_, and R_c_ can be obtained by subtracting R_w_ + R_o_ + R_p_ (104 Ω) from R_L_. The obtained C_i_, E_c_, and R_c_ were presented according to the number of field cycling in figure 3.

**
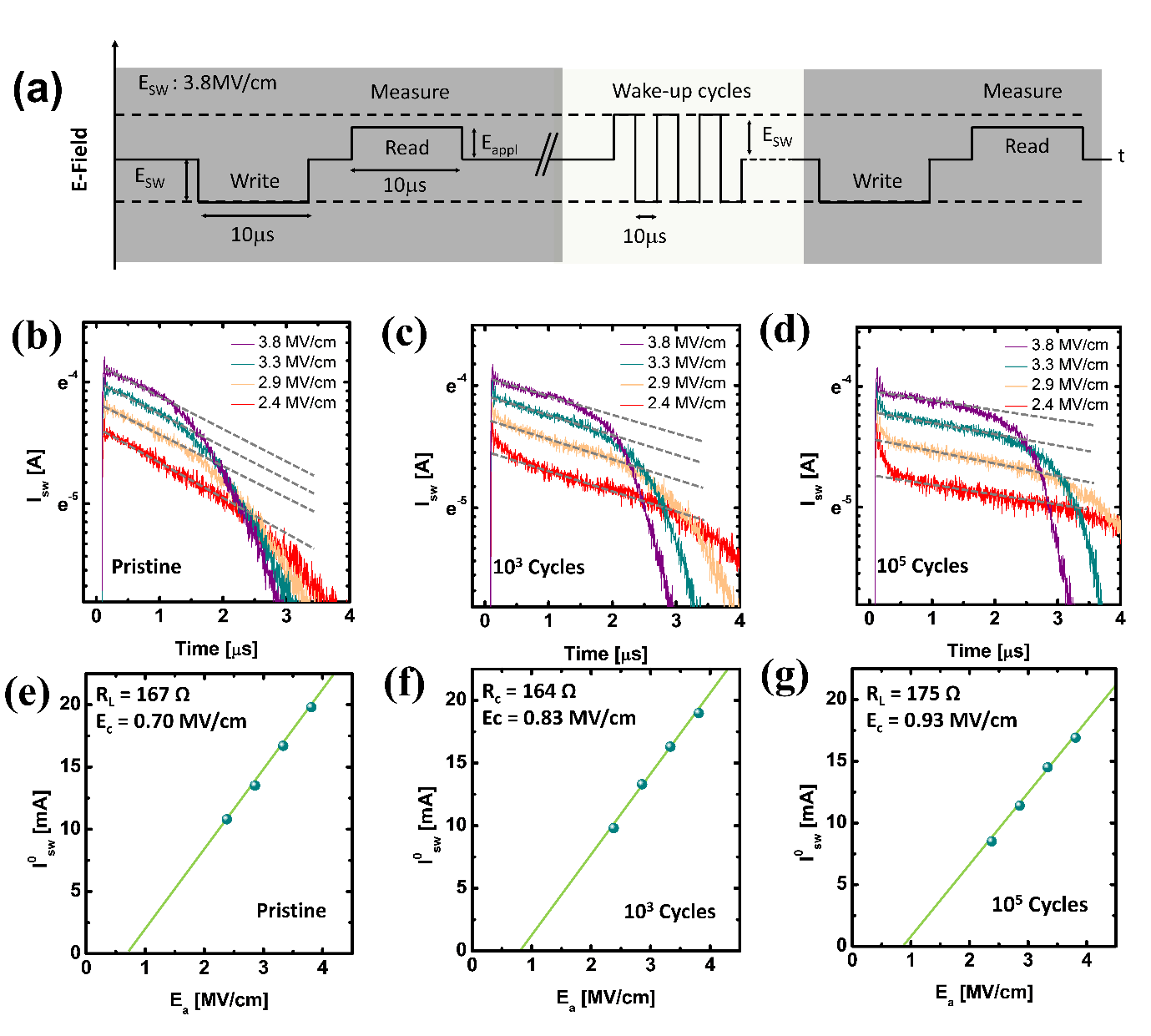
Figure S1.** (a) The schematic diagram for pulse application with time. The domain switching current transient–time curves of the TDMA HZO film with various applied electric fields, (b) in the pristine state, (c) after 10^3^ electric switching cycles and (d) after 10^5^ electric switching cycles, respectively. The initial current value (I^o^_sw_) where the switching starts as a function of the electric field and extracted genuine coercive field and contact resistance of the same film, (e) in the pristine state, (f) after 10^3^ and (g) 10^5^ fatigue cycles (at 3.8 MV/cm, 10μs), respectively
